# Optimally controlling nutrition and propulsion force in a long distance running race

**DOI:** 10.3389/fnut.2023.1096194

**Published:** 2023-05-18

**Authors:** Cameron Cook, Guoxun Chen, William W. Hager, Suzanne Lenhart

**Affiliations:** ^1^Research Triangle Institute (RTI) Health Solutions, Research Triangle Park, NC, United States; ^2^Department of Nutrition, University of Tennessee, Knoxville, Knoxville, TN, United States; ^3^Department of Mathematics, University of Florida, Gainsville, FL, United States; ^4^Department of Mathematics, University of Tennessee, Knoxville, Knoxville, TN, United States

**Keywords:** optimization, differential equation models, running, nutrition, metabolism, bioenergetics

## Abstract

**Introduction:**

Runners competing in races are looking to optimize their performance. In this paper, a runner's performance in a race, such as a marathon, is formulated as an optimal control problem where the controls are: the nutrition intake throughout the race and the propulsion force of the runner. As nutrition is an integral part of successfully running long distance races, it needs to be included in models of running strategies.

**Methods:**

We formulate a system of ordinary differential equations to represent the velocity, fat energy, glycogen energy, and nutrition for a runner competing in a long-distance race. The energy compartments represent the energy sources available in the runner's body. We allocate the energy source from which the runner draws, based on how fast the runner is moving. The food consumed during the race is a source term for the nutrition differential equation. With our model, we are investigating strategies to manage the nutrition and propulsion force in order to minimize the running time in a fixed distance race. This requires the solution of a nontrivial singular control problem.

**Results:**

As the goal of an optimal control model is to determine the optimal strategy, comparing our results against real data presents a challenge; however, in comparing our results to the world record for the marathon, our results differed by 0.4%, 31 seconds. Per each additional gel consumed, the runner is able to run 0.5 to 0.7 kilometers further in the same amount of time, resulting in a 7.75% increase in taking five 100 calorie gels vs no nutrition.

**Discussion:**

Our results confirm the belief that the most effective way to run a race is to run approximately the same pace the entire race without letting one's energies hit zero, by consuming in-race nutrition. While this model does not take all factors into account, we consider it a building block for future models, considering our novel energy representation, and in-race nutrition.

## 1. Introduction

**Figure 1:**
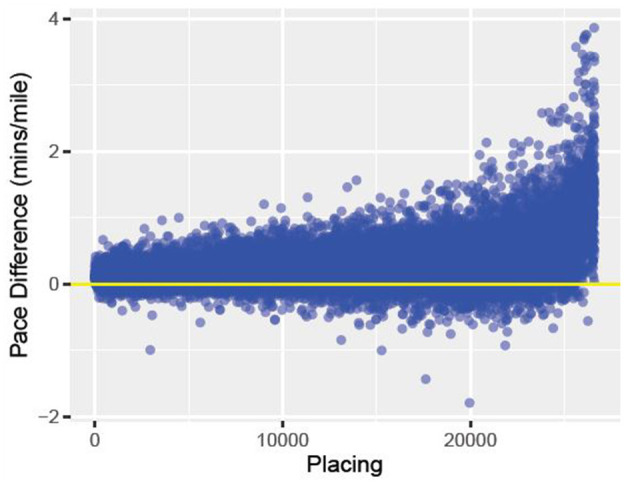
The above data comes from the 2015 Boston Marathon. We graphed finish placement on the *x*-axis and pace difference on the *y*-axis. Pace difference is the difference between the runners 5 km pace (min/mile) and the runners overall pace (min/mile) for the race. The horizontal line represents a difference of 0 between the two paces.

Running is one of the most popular forms of exercise. There are more than 275, 000 road races per year in the United States (1). At the marathon distance alone, there are over 500, 000 people a year in the United States who choose to race (www.runningtheusa.com). Some people run for fun while others choose to seriously compete, attempting to run the shortest time for a fixed distance race (2). Outside of sprints, pacing oneself to run the best possible race is crucial (3–5). If the runner starts the race too slow, they may not finish in as fast a time as expected. On the other-hand, if the runner goes out too fast, they may find themselves running out of energy, struggling to even finish the race (3–5). Determining the best pacing strategy for each individual runner is challenging, as a runner's optimal pace depends on several values that are individual to them, something this work aims to answer. Looking at data collected from timing mats in the 2015 Boston Marathon, Figure 1 plots how the runners placed vs. how their pace differed from their first 10 k and their overall pace. Amongst this elite field, one can see that, on average, the runners who placed lower had much higher pace variation than runners who finished in a shorter time (better placing).

While running the fastest race possible has been a goal for runners, it has also been a research topic for scientists for many years. Keller's model developed in the early 1970's, was the first to cast running an optimal race as an optimal control problem and has since been adapted and expanded by others such as Aftalion and Bonnans (6), Woodside (7), and Pitcher (8). All of the results from these works show the importance of pacing, but lack attention to the different energy systems. Kim et al. (9) applied whole-body metabolism models to exercise, but not to optimizing running race performance. Another factor that has not yet been considered in a runner model to date is, in-race nutrition as an energy source available to the runner. In order to avoid energy depletion during a long distance run, a runner can consume food. To meet this need there is a whole market of products for runners to use in order to deliver necessary fuel to the body quickly. As energy is currency for runners, including in-race nutrition in a model is essential in determining the optimal race for a runner tackling a long-distance race.

The goal of this work was to build a more dynamic runner model that better represents the body's energy systems, including energy allocation of fat and carbohydrate energy dependent on velocity, as well as in-race nutrition. We use this improved model to determine the minimum time it takes to run different length races (in particular the marathon distance race) by optimally choosing velocity and in-race nutrition consumption profiles through control of propulsion force and nutrition input. The inputs for the model are individualized such that an optimal race can be determined for any level of runner. We first discuss the physics, biochemistry, and other factors that can limit or enhance a runner's performance before presenting our model, and optimization techniques. Results and various simulations are presented, followed by discussion.

### 1.1. Background and groundwork from past models

There are many different factors that coaches and runners have to think about as a runner prepares for a race. They have to consider all the training needed to get the runner in the best possible condition for the race as well as all the in-race components (10). It takes time to train not just the muscles, but the body's energy pathways and metabolism. All the cumulative knowledge that exists today on developing the best training and racing plans began from understanding how the body transfers stored energy into mechanical work (11). Originally, scientists viewed exercise as the heat to mechanical energy transfer; however, biological energy transfer, or bioenergetics, was discovered to be better suited to describe the anaerobic and aerobic energy transfers due to chemical nature of the exchange (6, 11).

Keller (12) considered Newton's Second Law and oxygen supply, which was the first model of its kind, describing running races, and treats energy as available oxygen in the muscles per unit mass. Keller's first equation is the equation of motion:


(1) $$\begin{array}{l} \frac{dV}{dt}=f-\frac{V}{\tau } \end{array}$$

where *t* is time, *V*(*t*) is the instantaneous velocity, *f*(*t*) is the propulsive force per unit mass, $\frac{V}{\tau }$ is a resistive force per unit mass, and there is a constraint on the force, 0 ≤ *f* ≤ *f*_*max*_. Keller's second equation governs energy, as the oxygen balance equation for *e*_*an*_:


(2) $$\begin{array}{l} \frac{de_{an}}{dt}=\bar{\sigma }-fV \end{array}$$

where $\bar{\sigma }$ is the oxygen breathing and circulation rate in excess of that supplied in non-running state with the constraint *e*_*an*_(*t*) ≥ 0 for all 0 ≤ *t* ≤ *T* (12). Solving the optimization problem of minimizing the time to run a fixed by using only one energy type, Keller was able to obtain results very close to world records for shorter races. For example in the 100 m race, Keller's theoretical results garnered a time of 10.07 s while the world record at the time was 9.9 s, a percent error of 1.7% (12). As both anaerobic and aerobic processes happen in the human body, modelers like Aftalion and Bonnans (6), as well as others (7, 13) included both processes in their model. Woodside (7) included both processes and considered the body responding differently in longer races in his model by adding a fatigue factor for long distances; however, no consideration is given to what measures runners can take to combat fatigue. This work aims to address this and highlight the need to include two separate energies in a runner model.

### 1.2. Understanding the body's energy sources

When food rich in carbohydrates is consumed, it travels to the stomach where it is broken down and a product called glucose is released through a process called gluconeogenesis (14). In anaerobic metabolism the body uses only glycogen for energy through the creation of glucose (14). This process, called glycolysis produces energy quickly, but only two ATP (energy) molecules are obtained (14). On the other hand, the aerobic metabolism uses both fat and glycogen for energy and creates 38 ATP molecules through a more length process (14). The aerobic and anaerobic systems occur in separate cellular compartments (mitochondria and cytoplasm, respectively) and often at different rates, involve different reactants and products (14). Not only is the allocation of the two separate energy processes of interest, but also which fuel is being utilized (14). Glucose and fatty acids provide most of the fuel required for energy production in skeletal muscles during aerobic exercise whereas glucose is the main source of energy in anaerobic exercise (14). The body has significantly more energy available in the form of fat, but the rate of using this energy form cannot be increased at high exercise intensities when the anaerobic metabolism is the main mechanism (14). Thus, the body is mainly able to use fatty acids as an energy source at low levels of intensity (14). When not exercising, ~30% of the body's energy comes from glycogen and 70% from fat stores (14). These percentages shift when intensity increases, as does the number of calories being burned. Glucose is preferred as it is readily available and quickly metabolized, but is limited (14).

**Figure 2:**
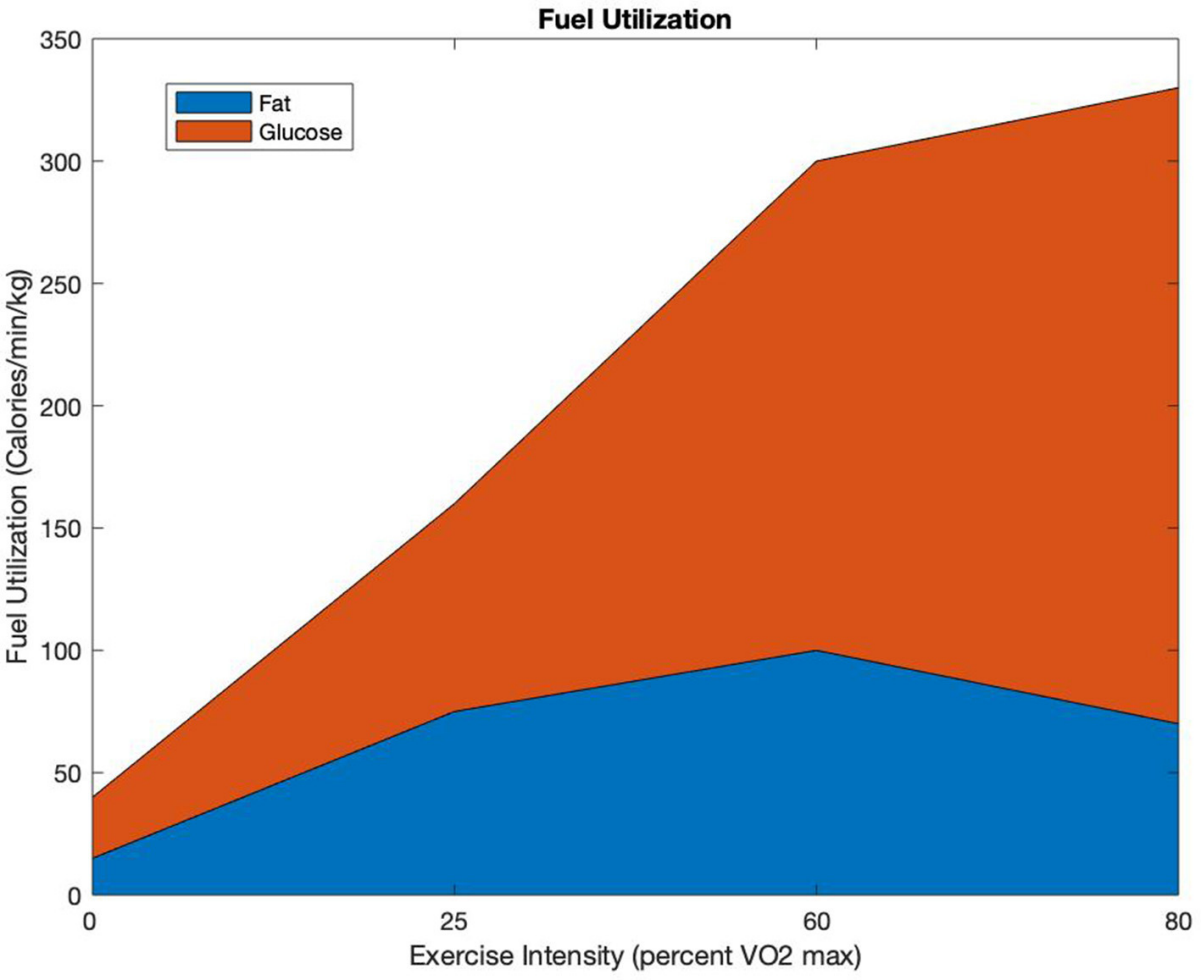
Fuel Utilization: allocation of calories per minute per kilogram used as a function of percent VO2max [adapted from (14)].

Figure 2 is a diagram showing fuel utilization between fat and glycogen as a function of percent VO2max. In Figure 2, at low percent of VO2max, the fuel utilization is low, and the percentage contribution from fat is substantial. As the percent of VO2max increases when running faster, the rate of fuel utilization increases and the percent contribution of fat decreases.

### 1.3. Quantifiable values that drive performance

Two quantifiable values that describe an athletes fitness are: VO2max, and VLamax (G Hillson, personal communication, April 21, 2021). Together, these two values are indicative of how a runner will perform, because they encompass the factors that make each athlete unique such as sprinting ability, endurance, training volume, gender, and experience. Both values play a role in describing an athletes energy expenditure and what metabolic system (aerobic or anaerobic) they are primarily using at a particular exertion level (14). Which metabolism is being used by the body is dependent on what percentage of one's current VO2 maximum (max) they are using, where VO2max is the maximum amount of oxygen you can utilize during exercise and is measured in milliliters of oxygen consumed in one minute, per kilogram of body weight (ml/kg/min) (14). This value usually ranges between 20–60 ml/kg/min with professional athletes holding values as high as 90 ml/kg/min (15). There are devices, formulas, and VO2max calculators (16, 17) that predict VO2max based on recent races and current level of activity.

In preparing for long distance races, runners are attempting to improve their VO2max through purposeful training, to allow the body to work at higher levels before needing to use the anaerobic system as the energy pathway (18). This means running at faster velocities without expending as much energy (18). When walking, people are between 15 and 30% of their VO2max, solely using their aerobic process, with the percentage increasing with exertion (14). When running a long-distance event, runners also mainly uses aerobic respiration and aims to stay at their aerobic threshold for the majority of the race, which is at about 60% VO2max (14). Runners should only use their anaerobic metabolism for a small portion of the race, as operating at such an intensity cannot be sustained for very long (14). The anaerobic system is activated when one is between 75 and 85% of their VO2max (14). During this time, the aerobic system is still used, but at a lower rate, until the runner reaches 100% of their VO2max (14). At that point, the anaerobic system is the only energy system being used as the runner is functioning at a level where they have used up all available oxygen and need an energy system that does not require oxygen (14).

One negative consequence of using one's anaerobic metabolism as the primary energy system is the byproduct of lactate from the reactions in the muscles (19). The accumulation of lactate in the body causes fatigue in the muscles and the intensity of exercise has to be lessened in order for the body to clear the lactate (19). Thus, one's ability to clear lactate significantly impacts their performance (20). A runner's VLamax or lactate capacity, is the body's anaerobic power, or maximum ability to produce lactate (G Hillson, personal communication, April 21, 2021). The higher the VLamax, the worse the runner is at clearing lactate near threshold (20), (G Hillson, personal communication, April 21, 2021). Marathon runners want to have a low VLamax so that they can use more fat for energy and spare their carbohydrates (G Hillson, personal communication, April 21, 2021).

While VO2max is a good indicator of fitness, a runner's VLa max is what sets the professional runner apart from one another (G Hillson, personal communication, April 21, 2021). Runners with a VO2max of 65 or with a VO2max of 80, could still have identical optimal races, depending on their VLamax. It doesn't matter if a runner has a high VO2max if they aren't able to access all of that oxygen (G Hillson, personal communication, April 21, 2021). In conclusion, a long-distance runner wants to train their body to have a high VO2max and a low VLamax (G Hillson, personal communication, April 21, 2021). These two values together are comprehensive in their ability to determine a runner's potential (G Hillson, personal communication, April 21, 2021).

### 1.4. Nutrition is necessary

When running races such as the marathon, one must consider pacing strategies (3–5). The runner must not only consider oxygen availability, but how much energy the body has stored (14). To optimally run these races, runners are mainly using the aerobic system, but their intensity with corresponding percentage of the VO2max is high enough for the body to use glucose as energy, causing them to still burn through the limited supply of glycogen. Runners of different levels, masses, running at relatively different VO2 values, burn through this glycogen at different rates; however, it is commonly accepted that on average runners burn just over 100 kilocalories per mile, meaning their stores will be depleted after about 20 miles or between 1.5 and 2 hours (14, 21). When these stores are depleted, the runner is forced to slow down significantly or stop running altogether and walk (22, 23). As only one person to date has completed the marathon in under 2 h, this is a major problem that long-distance runners must combat. In the 1960's, work by Burke et al. (24) confirmed that blood glucose concentrations were linked to fatigue and that eating hard candies during a race prevented weakness and fatigue during a race (21). Carbohydrates are digested in the small intestine and converted into glucose. Glucose is stored mainly in the muscles and the liver as glycogen, a chain of multiple glucose residues, but is also available for immediate use if necessary (14). The body can store about 600 grams of glycogen, with 500 grams stored in the muscles and 100 grams stored in the liver (14), totaling 2,400 Kilocalories of glycogen stores in the body.

During a long distance running race when glycogen stores are depleted, ingested or exogenous carbohydrates are quick sources of energy for the muscles, available once absorbed by the muscles from the blood. Runners typically consume gels, which are a concentrated dose of glucose throughout long distances races. Most runners solely consume gels, that contain ~25 grams of carbohydrates (100 Kilocalorie).

Taking in nutrition during the race allows a runner to move longer before glycogen stores are depleted and they've reached some anaerobic energy threshold where they must walk (23). It is known that the muscles absorb plasma glucose at a maximal rate of 1–1.7 g/min (14, 25, 26) depending on the sugar mixture. This means that while it may only take 3–5 min for some of the carbohydrates from a 100 calorie intake to reach the muscles, it can take ~25 min for all of the carbohydrates from the package to be absorbed. While there is no limit to how many carbohydrates a runner can ingest, too many carbohydrates consumed in a short time will result in digestive discomfort, forcing the runner to slow down (23).

## 2. Methods

We build on the current models by adding in some novel terms as well as completely reformulating the energy equation. Our overall goal is to determine the best nutrition and pacing strategy to use when running a marathon (or other long distance race) in order to finish in the shortest amount of time. Our first objective, is to develop a runner model that takes into account fuel allocation depending on percentage VO2 max, fuel intake (in-race nutrition), as well as the force which is applied to the ground by the runner, by using a system of differential equations. Next, we would like to cast this as an optimal control problem to determine the optimal velocity and in race nutrition consumption profiles through control of propulsion force and nutrition input.

### 2.1. The model

**Figure 3:**
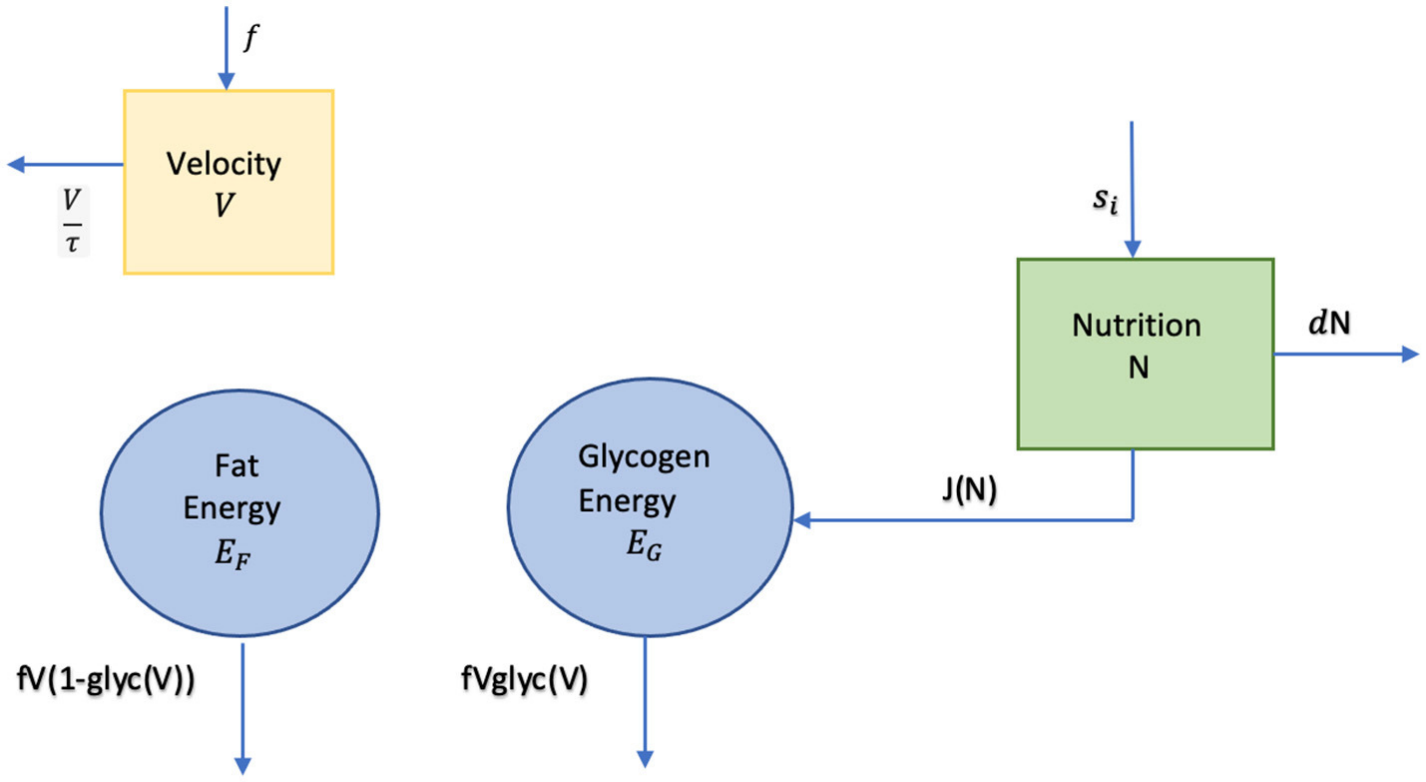
Energy-nutrition-velocity diagram.

Our system of ordinary differential equations for: *V*, Velocity, *E*_*F*_, Fat Energy, *E*_*G*_, Glycogen Energy, *N*, Nutrition where *V* will be measured in meters/ min, *E*_*F*_ and *E*_*G*_ in KJ/Kg, *N* in KJ, and our control force *f*(*t*) in meters/min^2^, are given by:


(3) $$\begin{array}{l} \frac{dV}{dt}=\;f\left(t\right)-\frac{V}{\tau } \end{array}$$

(4) $$\begin{array}{ll} \frac{dE_{G}}{dt}=\; & c_{3}j\left(N\right)-af\left(t\right)Vglyc\left(V\right) \end{array}$$

(5) $$\begin{array}{l} \frac{dE_{F}}{dt}=\;-af\left(t\right)V\left(1-glyc\left(V\right)\right) \end{array}$$

(6) $$\begin{array}{l} \frac{dN}{dt}=\;s\left(t\right)-dN-j\left(N\right) \end{array}$$

where $a=\frac{1}{1000}$. Figure 3 is a diagram of our system, including the velocity compartment. Velocity is not directly connected to any of the other compartments via energy transfer, but its impact on the two energy compartments can be seen in Equations (4) and (5). Our nutrition intake strategy is the fuel source function, *s*, entering in the nutrition compartment.

In Equation (3), there is a propulsive force per unit mass *f*(*t*), and $\left|\frac{V\left(t\right)}{\tau }\right|$ is a resistive force per unit mass, with τ as a constant of proportionality. The initial condition for this equation is: *V*(0) = 0, as we start from rest, and we have a constraint on the force, 0 ≤ *f*(*t*) ≤ *f*_*max*_. This is the same equation that Hill, Keller, and others used for velocity (6, 7, 13, 27, 28).

As energy is available from two different processes in the body, anaerobic and aerobic metabolisms, we consider two energy sources. Thus, our total energy, *E*, can be written as *E* = *E*_*F*_+*E*_*G*_. The human body has large stores of fat for use as energy, but the body prefers to use glycogen when moving at higher rates; thus, a higher percentage of fuel usage comes from fat at lower velocities. As runners typically take gels that are mainly sugar (not a significant source of fat), we assume there is no input into the fat energy compartment. The body expends energy at a rate, *f*(*t*)*V* (work done). As we are allocating the energy usage between the two sources, our fat energy differential Equation (5), and glycogen energy differential Equation (4) have glycolitic function, *glyc*(*V*), a fuel allocation function of one's glycogen energy through the anaerobic and aerobic pathways dependent on the quotient of one's instantaneous velocity, *V*, and their velocity, *VVO*2*max*, at 100% VO2max, written $\frac{V}{VVO2max}$. Thus, 1 − *glyc*(*V*) accounts for the fuel allocation function of one's fat energy through the aerobic pathway dependent on velocity compared to one's velocity at 100% VO2max.

It is difficult to know one's current percentage VO2max; however, one could know a priori their velocity at 100% VO2max, a term known as VVO2max (29). Billat and Koralsztein (29) showed that one's VO2maxand their velocity at VO2max are linearly related. As velocity is more easily computable, this relationship is used in our model for determining the allocation of energy at different velocities compared with one's velocity at VO2max. To approximate *glyc*(*V*), we first described it as a piecewise continuous function on intervals of $\frac{V}{vvmax}$ based on Figure 2 from (14) and then approximated that function by a function that smooths the points where the derivatives do not exist (described in detail later).

Equations (4) and (5) have a convex combination of this function and the fat allocation function of 1 − *glyc*(*V*), as the total work rate must equal *f*(*t*)*V*. We obtained the piecewise graph for *glyc*(*V*) from Figure 2 as well as from the literature (14). Figure 2 gives a good estimate of how the body uses fat energy vs. glycogen energy.

**Figure 4:**
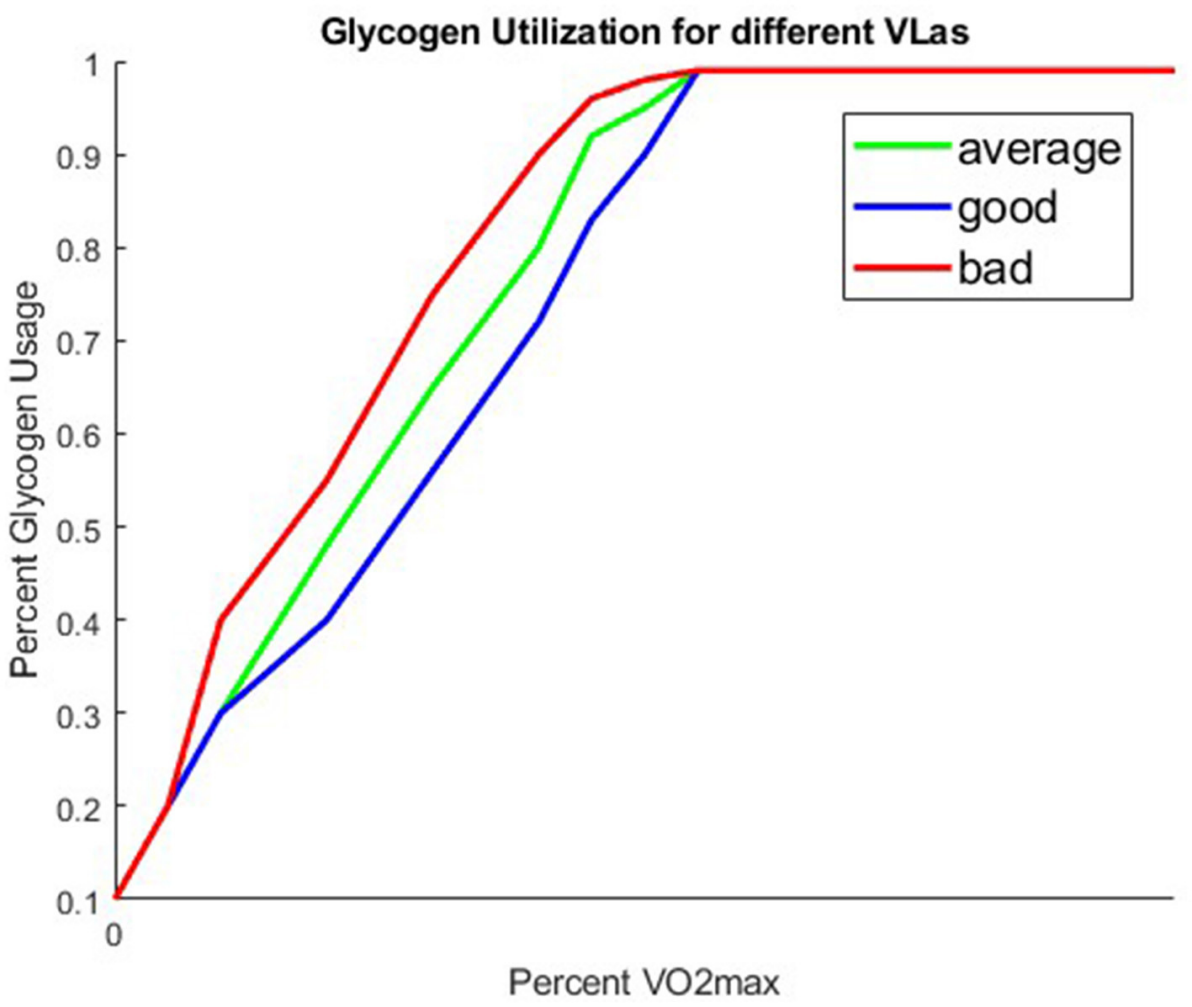
Three different glyc functions corresponding to runners with a good, average, or bad VLa.

We assume that the anaerobic pathway is not used until after 60% VO2max, and that the energy from fat linearly decreases to 0 by 100% VO2max at which point the anaerobic system, and thus the glycogen compartment, is solely used. Figure 4 is a graph of three different possible *glyc*(*V*) functions that we obtained from our Figure 2.

Recall, that while what percentage of one's VO2maxat which they are running is explicitly in the *glyc*(*V*) function, VO2max is not the only value that effects our fuel utilization. Lactate capacity (VLa), also impacts one's fuel utilization as described earlier. We account for differences in VLa, by considering different structures for our fuel utilization function, *glyc*(*V*). The function is still dependent on percent VO2max, but varies in fuel utilization at particular percent VO2maxvalues. Figure 4 shows three different *glyc*(*V*) functions corresponding to three different lactate capacities. We use these three different glyc structures to represent runners who have a low VLa (good), an average VLa (avg), and a high VLa (bad). The runners with a low VLa (good) are able to run at a faster pace than those with a higher VLa without accumulating as much lactate in their muscles. The VLa is a feature that sets the best professionals apart from one another.

In Equation (4) for glycogen energy there is a source term with *j*(*N*) that comes from the nutrition differential Equation (6). Our *j*(*N*) term is a nutrition consumption function increasing energy available in the muscles in the form of glycogen, but at a bounded rate: *j*(*N*) = *c*_4_*N* with rate constant $c_{4}=\frac{1}{m}$, the inverse of the runners mass. Our initial conditions for our two energy equations are *E*_*G*_ = 144 Kilojoules per unit mass, assuming the runner has full glycogen stores, and *E*_*F*_(0) = 3, 439 Kilojoules per unit mass, dependent on the runner's body fat percentage. Both of the energies must stay non-negative, and dictate the choices of *f*(*t*) and the corresponding V. Thus, in particular, if *E*_*G*_ = 0, *f*(*t*) = 0. This is not optimal and would be avoided in an optimal race until the very end.

Further, in our nutrition energy differential equation, Equation (6), *s*(*t*) is a source term from nutrition input such as a gel. Each gel is roughly 100 Kilocalories (4.18 Kilojoules). The addition of the source term slows the rate at which *E*_*G*_ decreases. The −*dN* term in the nutrition differential equation represents the nutrition used for basic bodily function, not available to the muscles for energy usage. The initial condition for this equation is *N*(0) = 0 as we assume there is nothing in this compartment when the race starts. Model parameters can be found in the results section in Table 2.

### 2.2. Optimal control

Our second goal is to determine the best strategy to use when running a long distance race in order to finish in the shortest amount of time. Casting this as an optimal control problem, we could think of this as a problem of maximizing the distance over a fixed time interval or equivalently [proved in (6)], as a problem of minimizing time over a fixed distance. Solving the minimum time problem requires an extra isoperimetric constraint; thus, we choose to solve the maximum distance problem. We have two controls in our problem: propulsive force, *f*(*t*), and fuel intake *s*(*t*).

We will determine the optimal control *f*(*t*) for each of the intake strategies and then optimize over those intake strategies. As there are many philosophies within the running community about how often you should take nutrition during a race, we test 15 different nutrition strategies, *S* = {*s*_0_, *s*_1_, *s*_2_, …, *s*_15_}, shown in Table 1. For the maximum distance problem, assuming we have a nutrition strategy, *s*_*i*_, we maximize the objective functional:


(7) $$\begin{array}{l} J_{i}(f)=\int_{0}^{T}V(t)dt \end{array}$$

for *s*_*i*_ ∈ *S* (a finite set of nutrition strategies), where


$$S=\left\{s_{1},s_{2},...,s_{N}\in L^{2}\left(0,T\right)|0\leq s_{i}\left(t\right)\leq s_{max},a.e.,i=1,2,...,N\right\}$$

we determine an optimal control depending on *s*_*i*_, and then optimize over our set *S*. We obtain continuous velocity and energy profiles corresponding to our control force and then optimize over discrete *s*_*i*_. We solve the following with fixed *T*:


(8) $$\begin{array}{l} \underset{S}{\max}\underset{A_{i}}{\max}J\left(s_{i},f\right)=\underset{S}{\max}\underset{A_{i}}{\max}\int_{0}^{T}V\left(t\right)dt \end{array}$$

with bounded controls: 0 ≤ *f* ≤ *f*_*max*_, 0 ≤ *s*_*i*_(*t*) ≤ *s*(*t*)_*max*_, and control set


$$U_{i}=s_{i}\times \left\{f\in L^{2}\left(0,T\right)|0\leq f\left(t\right)\leq f_{max},a.e.\right\}$$

subject to state constraints: 0 ≤ *E*_*F*_(*t*) 0 ≤ *E*_*G*_(*t*), for *t* ∈ [0, *T*]

initial conditions: *V*(0) = 0, *E*_*F*_(0) = *K*, *E*_*G*_(0) = 144, *N*(0) = 0,

and state Equations (3)–(6).

**Table 1:** Scenarios of nutrition intake during a marathon race.

*s*_0_,*s*_1_,*s*_2_,*s*_3_, *s*_4_,*s*_5_,*s*_6_, *s*_7_	100 Calorie gels spread evenly 0, 1, 2, 3, 4, 5, 11, and 24 times throughout the race.
*s*_8_	One 100 Calorie gel taken toward the beginning of the race.
*s*_9_	One 100 Calorie gel taken toward the end of the race.
*s*_10_	Four 100 Calorie gels: two taken early in the race, two taken toward the end of the race.
*s*_11_	Four 200 Calorie gels spread evenly throughout the race.
*s*_12_, *s*_13_	250 Calorie gels spread evenly 2, 4 times throughout the race
*s*_14_	Ten 50 Calorie gels taken evenly throughout the race.
*s*_15_	One 250 Calorie gel taken, one 100 Calorie gel taken, one 250 Calorie gel taken.

Our admissible control set for *s*_*i*_ ∈ *S* is


$$A_{i}=\left\{\left(s_{i},f\right)\in U_{i}|E_{F}\left(t\right)\geq 0,E_{G}\left(t\right)\geq 0,\;\text{for}t\geq 0\right\}$$

Note that *V*(*t*) and *N*(*t*) are non-negative, from Equations (3) and (6). The proof of the existence of an optimal pair $\left(s_{i}^{*},f^{*}\right)$ can be found in the Supplementary material and (30).

Using Pontryagin's Maximum Principle (PMP) (31), we were able to understand some features of the optimal control f^*^ with corresponding $s_{i}^{*}$. For most of the time interval, the optimal control is singular, meaning that the objective functional is flat with respect to the control. See the Supplementary material for some ideas about the necessary conditions satisfied by the optimal control f^*^ given a nutrition strategy $s_{i}^{*}$. This “singular" feature led us to our numerical solution algorithm as explained in the methods section (32). We discretize the system in order to implement an approximate solution to the optimal control problem, including a penalization term known to reduce the noise in optimal control problems with singular solutions (33).

### 2.3. Discretization

Over time, optimal control problems have been solved and approximated using many different numerical techniques. There are many numerical examples in mathematical biology that use the Forward Backwards sweep method (34, 35). Programs such as GPOPS and PASA have been developed to handle particular types of optimal control problems (36, 37). MATLAB has a minimization tool, fmincon, that is built to handle a variety of optimization problems. We tried to use the forward backwards sweep method, the packages GPOPS and PASA, as well as fmincon in the continuous setting; however, none of these methods were robust enough to handle this particular problem. Thus, we discretized our problem and used fmincon, inputting our differential equation system in through equality constraints.

We began the discretization of our optimal control problem by partitioning our time interval, [0, *T*] using M+1 equally spaces nodes, 0 = *t*_0_ < *t*_1_ < ⋯ < *t*_*M*_ = *T*. We used a left rectangular approximation for the objective functional, obtaining the maximization problem:


(9) $$\begin{array}{l} \underset{f}{\max}J\left(f\right)=\underset{f}{\max}\left[\overset{M-1}{\underset{k=0}{\sum }}hV_{k}\right] \end{array}$$

where $h=\frac{T}{M}$ with bounded controls: 0 ≤ *f*_*k*_ ≤ *f*_*max*_ = 36, 000 meters/min^2^, and *f* = (*f*_0_, …, *f*_*M* − 1_), with *s* = (*s*_1_, …, *s*_15_) ∈ *S*, and 0 ≤ *s*_*i*_(*t*_*k*_) ≤ *s*_*max*_ for all 0 < *k* < *M* − 1 and with initial conditions: *V*_0_ = 0, *E*_*F*, 0_ = *K*, *E*_*G*, 0_ = 144, *N*_0_ = 0.

Next we use a forward Euler approximation for the state equations and obtain:


(10) $$\begin{array}{l} V_{k+1}=V_{k}+h\left(f_{k}-\frac{V_{k}}{\tau }\right) \end{array}$$

(11) $$\begin{array}{l} E_{F,k+1}=E_{F,k}+h\left(-af_{k}V_{k}\left(1-glyc\left(V_{k}\right)\right)\right) \end{array}$$

(12) $$\begin{array}{l} E_{G,k+1}=E_{G,k}+h\left(c_{3}j\left(N_{k}\right)-af_{k}V_{k}\left(glyc\left(V_{k}\right)\right)\right) \end{array}$$

(13) $$\begin{array}{l} N_{k+1}=N_{k}+h\left(s\left(t_{k}\right)-dN_{k}-j\left(N_{k}\right)\right) \end{array}$$

We also now have the discretized version of *J*(*N*_*k*_): *j*(*N*_*k*_) = *c*_4_*N*_*k*_ . We discretized our glyc function, *glyc*(*V*_*k*_), by using a spline interpolator in MATLAB, described in the next section, and dropped in each *i*^*th*^ nutrition scenario, *s*_*k*_, corresponding to the time points each nutrition strategy designated. To optimize our system, while satisfying the constraints, we use fmincon as the minimization solver on the discretized system, using a left-rectangular integral approximation for the objective function.

“Fmincon is a gradient-based method that is designed to work on problems where the objective and constraint functions are both continuous and have continuous first derivatives" (38), but due to our constraints and nutrition input, it was helpful to write it as a discrete system. Fmincon is set to accept: the objective function, a starting guess for the control vector, X0, inequality linear constraints, equality linear constraints, lower and upper bounds for the state variables, non-linear inequality and equality constraints, as well as options that allow the user to change the MATLAB settings for various features or provide the system with more information. Our discretized objective function was originally


$$J=-\delta \overset{T-1}{\underset{i=1}{\sum }}V_{k}$$

where *V*_*k*_ occurs in our *X* vector as entries *X*(3*n*+1:4*n*) and $\delta =\frac{T}{M}$ where *T* is the length of the race and *M* is the number of mesh points, including *t* = 0, but we will modify this objective function to include minimizing the total variation in *f*. In order to modify the objective function to include minimizing the total variation, we added two additional state variables, *ι* and *ζ* (39), such that the variation in two time points in *f* is written as


f(i+1)-f(i)=ζ(i)-ι(i) .

Our starting guess vector, *X*0, that gives an initial placeholder for our state vector was: $X0=\left[\overset{T}{\overset{︷}{36000\;\ldots 36000}}\;\overset{6T-2}{\overset{︷}{0\;\ldots 0}}\right].$ Note that each state vector variable is a column vector of length (*T* × 1) and the two vectors used for variation penalization, *ζ* and *ι* are of length (*T* − 1) × 1 due to their structure. Our initial condition vector was: [*f*(1) *E*_*F*_(1) *E*_*G*_(1) *V*(1) *N*(1) *ζ*(1) *ι*(1)] = [36000 3439 144 0 0 0 0] . As we do not have any inequality constraints and our equality linear constraints were written as: *A*_*eq*_*x* = *b*_*eq*_, where *Aeq* is a *n* × *n* matrix, *x* is our solution column vector of length *n* × 1, and *beq*, of length *n* × 1 is the righthand side of our equality linear constraints.

As the velocity and nutrition equations are linear, we input those as our linear equality constraints. Our two energy equations are non-linear and thus are input as non-linear equality constraints. One important feature for our problem is our energy constraints, *E*_*G*_(*i*) ≥ 0 and *E*_*F*_(*i*) ≥ 0 for every *i* ∈ [0, *T*]. With our problem set up in its current format, we are able to simply input our lower bounds for our fat and glycogen energies as 0. We input the runners physical force, velocity, and energy capacities as appropriate upper bounds. We also give our variables appropriate initial conditions for the runner's initial fat and glycogen energies, 0 velocity, and 0 in race nutrition.

In our glycogen energy equation we have a function we call “glyc" and its counter part “1-glyc" in our fat energy equation. We approximated these from a fuel utilization. Figure 4 adapted from one in Stipanuk and Caudill's textbook (14), by creating two vectors of points where one vector represents the percent VO2maxand the other represent the percent glycogen used. To smooth out our glyc function, we used a function in MATLAB called spline. Spline is a cubic piecewise polynomial interpolator that is continuous and twice differentiable everywhere. It takes the two vector of points you supply it with and creates *n* − 1 cubic polynomials that connect at the supplied *x* vector given.

One common issue in control problems where the control has a singular sub-arc is variation. Ding and Lenhart (40) as well as Caponigro et al. (41) both penalized their original objective functional to regularize the chattering. In order to obtain a reasonable trajectory for the runner, we chose to penalize the objective functional by adding a term to our objective functional that bounded the total variation (33, 39). This technique, developed by Hager and Aghaee (33), has been shown to reduce the noise in optimal control solutions. Adding this penalty, not only amends the objective function to include penalizing for variation, it adds a 5th constraint to our problem. By adding this penalty we are decreasing the total variation in the solution trajectory. We can express the total variation, V(f), in the control (39) as:


$$V\left(f\right)=\sup\overset{N-1}{\underset{i=0}{\sum }}|f\left(i+1\right)-f\left(i\right)|$$

where the supremum is being taken over all possible partitions of our time interval. Due to the difficulty of differentiating absolute value functions we decompose the absolute value term, using two new *T* − 1 vectors *ζ*(*i*) and *ι*(*i*), whose entries are non-negative. Each entry of *ζ*(*i*) and *ι*(*i*) will be defined as


f(i+1)-f(i)=ζ(i)-ι(i)

We can think of this decomposition as satisfying the following two conditions (39):

If *f*(*i*+1) − *f*(*i*) > 0, then ζ(*i*) = *f*(*i*+1) − *f*(*i*) and *ι*(*i*) = 0;

If *f*(*i*+1) − *f*(*i*) ≤ 0, then ζ(*i*) = 0 and *ι*(*i*) = − (*f*(*i*+1) − *f*(*i*)),

where *ζ*(*i*) ≥ 0, *ι*(*i*) ≥ 0 and either *ζ*(*i*) = 0 or *ι*(*i*) = 0

Appending this penalty to the objective function we have:


$$J=-\delta \overset{T-1}{\underset{i=1}{\sum }}V\left(i\right)+p\overset{T-2}{\underset{i=1}{\sum }}\left(\zeta \left(i\right)+\iota \left(i\right)\right)$$

and resulted in the extra linear equality constraint


ceq5(i)=-f(i+1)+f(i)+ζ(i)-ι(i),

The coefficient *p* of our bounded variation scales the degree to which we penalize variation. If *p* is large, there is a more emphasis being placed on minimizing the variation of the control variable, force.

## 3. Results and discussion

### 3.1. World record optimal results

We found that using a direct discrete optimization without adjoint functions, fmincon, was the most efficient approximation method and captured all the dynamics. Table 2 shows the constants and coefficients with their scientific meaning and their units. Five parameters that can be chosen depending on the individual are: *E*_*G*_(0) (Initial glycogen energy), mass, VLa type, nutrition uptake rate, *c*_4_, and VVO2max, shown at the end of Table 2.

**Table 2:** Parameters used for world record holder.

**Parameter**	**Value**	**Unit**	**Meaning**
*T*	120	minutes	Length of race
*M*	*T*+1	minutes	Number mesh points including *t* = 0
τ	1/60	min	Internal resistant force constant
*d*	0.005	min^−1^	Loss of nutrition
*sm*	1/3, 600	(seconds)^−2^	Seconds to minutes conversion
*c*_3_	1/*m*	kg^−1^	Mass conversion constant
*p*	0.5	unitless	Variation penalty coefficient
*a*	1/1, 000	kilojoules/joules	Unit conversion constant
δ	120/121	T/M	Discretization parameter
*m*	55	kilogram	Runner mass
*E*_*G*_(0)	144	kilojoules/kg	Initial energy at start of race
*c*_4_	1/6	min^−1^	Nutrition uptake rate
VVO2max	402	meters/min	Velocity at 100% VO2max
VLa type	Good	Unitless	Good, average, bad in glyc function

We first chose to simulate the optimal race of the current world record holder. In the attempt to break the 2 h marathon barrier, experts collaborated to optimize a set of runners VLamax, VO2max, general running economy, nutrition plan, as well as pick the perfect race course for these runners. The parameter values for our simulation can be found in Table 2. During Eulid Kipchoge's race to beat the 2 h marathon barrier, his nutrition consisted of hydrogels by Maurteen. He consumed 100 g Carbs per hour (which would be four baked sweet potatoes in solid food for reference) (42), although there is no comment on his exact feeding regime. This amount of carbohydrates is pretty high for a runner to tolerate during a marathon due to the increased gastrointestinal movement in running. This number of carbohydrates is more in line with what cyclist would consume during their longer races. This is in contrast to the practice most runners follow of taking about 50 carbs per hour, an area of research that is continuously being studied by nutrition companies.

**Figure 5:**
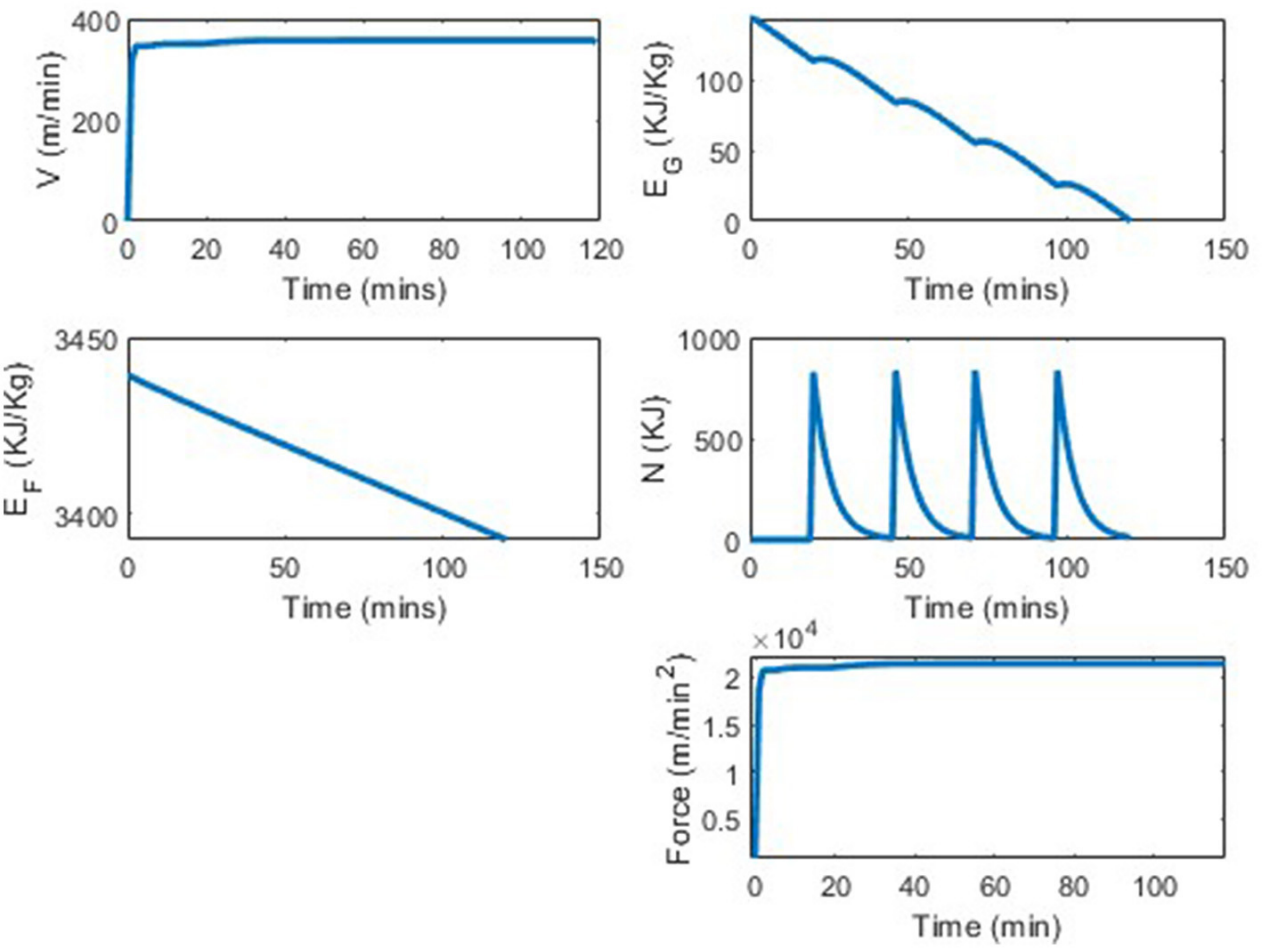
World record marathon simulation, with four 200 calorie gels.

While three different VLA types are shown in Figure 4, the “good" glyc was picked to reflect appropriate values for a professional (world record breaking) runner. Plots of the state and control variables are seen in Figure 5. In Figure 5 we see that the runner's velocity rapidly increases from 0 to reach an approximately constant velocity of 357 m/min, that they can maintain for the entirety of the race. The plot of the runner's propulsion force, similar to the runner's velocity plot, shows the runner's force quickly increasing from 0 m/min^2^ to a constant force of 2.14·10^4^ m/min^2^. The runner's fat energy, *E*_*F*_, decreases linearly, from 3, 439 KJ/Kg to 3390, nowhere near the energy constraint, *E*_*F*_ ≥ 0. The runner's glycogen energy decreases from its initial value of *E*_*G*_(0) = 144 KJ/Kg to its final value of ~0 KJ/Kg. We see bumps in this subplot corresponding to the nutrition fueling. In this simulation we assumed that the runner took four 200 calorie gels at *t* = 20, 46, 71, 97, which can be seen in the nutrition subplot in Figure 5, and the runner took 120 min to complete the distance.

The VVO2max was set to be 402 meters per minute, as this is a reasonable assumption for a world class runner. The fastest marathon ever run thus far is 1:59:40. In the simulation from our model, the runner completes 42.5 kilometers in 120 min, which is equivalent to the runner running a 1:59:09 marathon. This is only a 0.4% difference between the current world record and the results from this model. This difference in average pace would be the runner running 4:33 min per mile instead of 4:34 min per mile. Note, that although our results are extremely close to the current world record, parameters could be changed to address a runner having an even better VLa, or better running economy. We also didn't explicitly have “shoe type" in our model but a better shoe could result in better running economy which would result in a better VVO2max. It is believed that the world record time will continue to get faster over time. For an optimization runner model, the results from the world record marathon run were the most ideal comparison; however, to see the model's robustness, we vary a variety of parameters and consider several potential scenarios. We first considered the scenario of no in-race nutrition consumed, then various nutrition scenarios, before concluding by varying other inputs.

### 3.2. Results: varying runner dependent parameters

A runner's in-race nutrition, VLamax, and their VO2max are the driving factors behind a runners performance, and thus it is important to see how changing these impacts the results. First, analyzing nutrition, there are several nutrition strategies over which we are optimizing, three of which include: simulating Kipchoge's known race intake, a standard intake strategy used by average runners, as well as taking no nutrition labeled *s*_0_, from Table 1, as some runners complete marathons without taking in-race nutrition. The parameters used for the 15 different nutrition strategies are all the same as those used in Table 2, except, *T* = 135, which also changes $\delta =\frac{135}{136}$.

**Figure 6:**
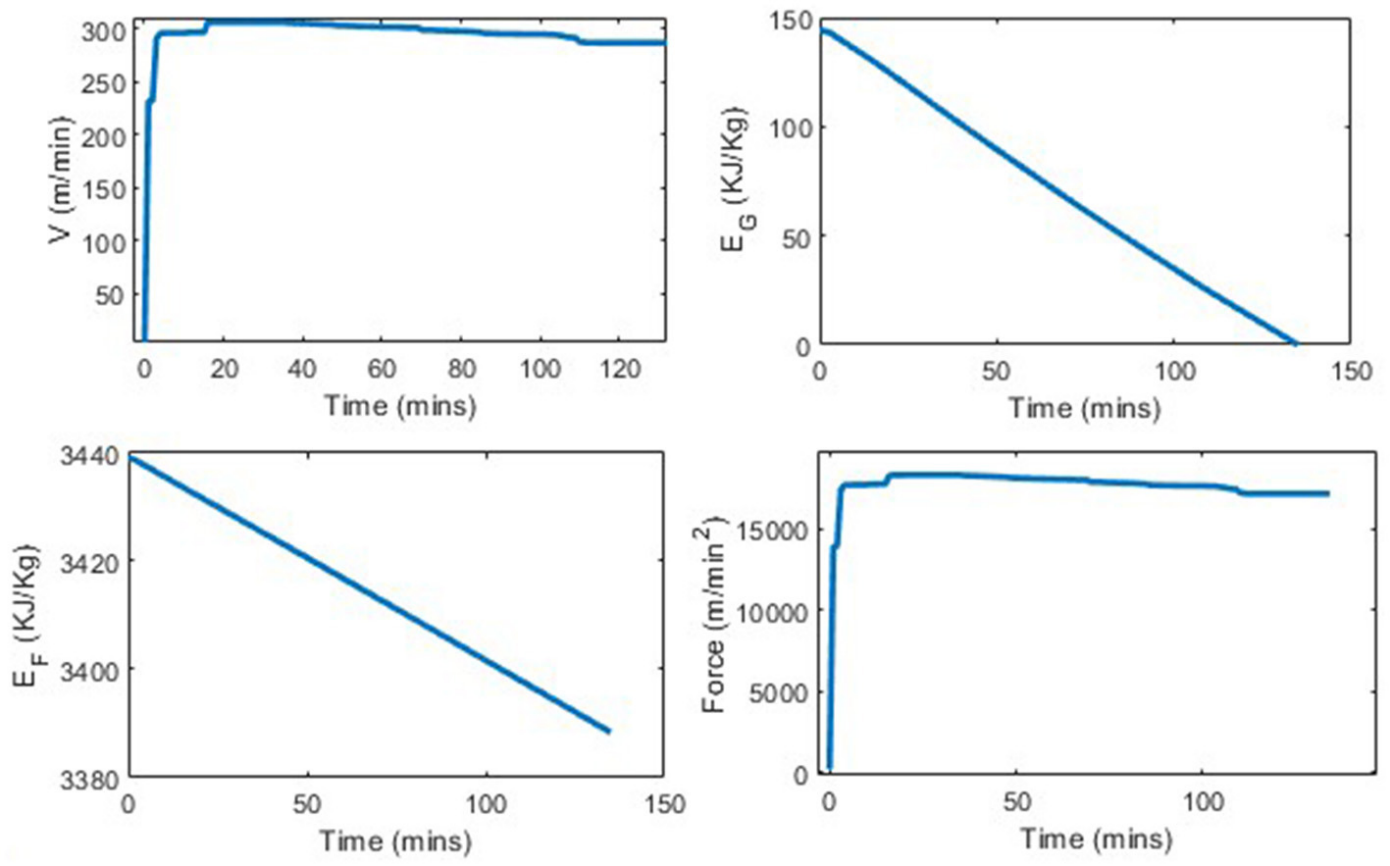
Marathon simulation with three states and optimal force with no gels.

**Figure 7:**
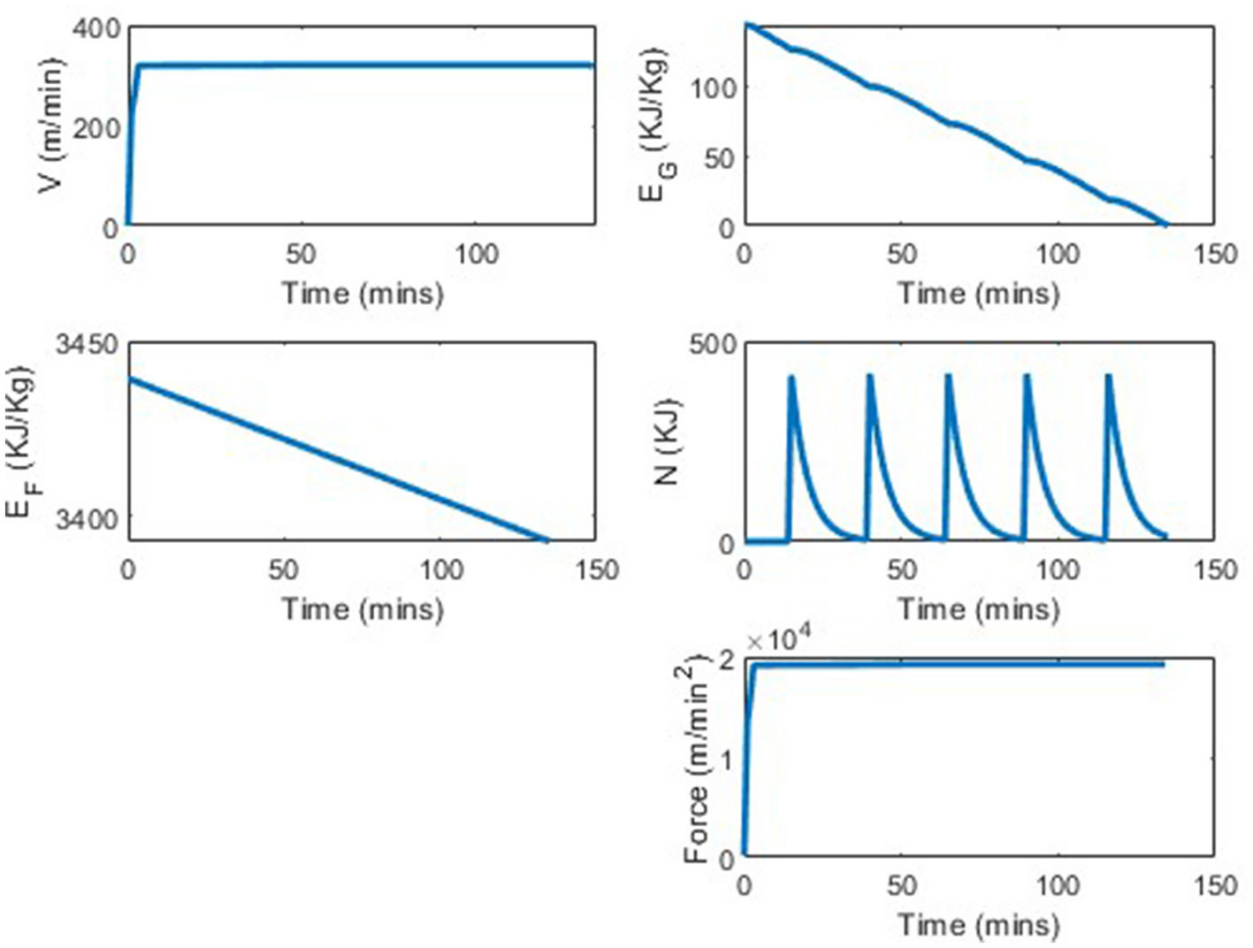
Marathon simulation with four states and optimal force with five 100 calorie gels.

We analyze the percentage improvement from using the determined strategy vs. consuming no carbohydrates. As stomach sensitivity to food intake during the race has not been included in this model yet, the results skew in favor of taking as much nutrition in as possible. Table 3 shows the distance completed with each of the tested strategies. In comparing a strategy of taken 0 nutrition vs. five 100 cal gels, the runner is able to run between 0.5 and 0.7 kilometers further per each additional gel 3, which translates to ~1.5% increase in distance per gel. By taking five 100 calorie gels instead of taking no nutrition, the runner has a 7.75% improvement in performance. In Figures 6, 7 we see similar strategies in the force and velocity subplots; however the propulsion force and therefore the velocity the runner is able to maintain, is significantly higher for the runner who took five 100 calorie gels than for the runner who took no nutrition.

**Table 3:** Total distance achieved with different nutrition strategies.

**Strategy**	**Distance (km)**	**Strategy**	**Distance (km)**
*s*_0_	40.0	*s*_8_	40.7
*s*_1_	40.7	*s*_9_	40.5
*s*_2_	41.4	*s*_10_	42.5
*s*_3_	42.0	*s*_11_	45.1
*s*_4_	42.6	*s*_12_	43.2
*s*_5_	43.1	*s*_13_	45.6
*s*_6_	46.0	*s*_14_	43.1
*s*_7_	52.9	*s*_15_	43.7

We were also interested if there would be any differences in performance if the runner took in the same amount of calories, but distributed differently. In *s*_5_, where the runner took in 500 calories by taking five 100 calorie gels, the distance achieved was 43.1 km. In *s*_12_ where the runner takes in two 250 calorie gels the distance achieved was 43.2 km. Lastly, in *s*_14_ where the runner consumes ten 50 calorie gels, the runner traveled 43.1 km. Regardless of the way in which the runner consumes the same amount of calories, they travel relatively the same distance. Perhaps this would be different if there were a mechanism in the model that discouraged eating too much at once or eating later in the race.

A typical marathon runner, may consume five 100 calorie gels throughout a race. This is in contrast to the intake of professionals who are taking up to 100 carbohydrates an hour, translating to 400 calories an hour. A runner finishing the marathon in about 2 h would take in 800 calories, which is simulated with strategy *s*_8_, in Figure 5. In this simulation the runner is able to run 1.5 km further when consuming 800 calories instead of 500 calories, an improvement of 3.5%. In both simulations we see similar trajectories; however, the runner in simulation *s*_8_ is able to run at a higher average speed throughout the race, yielding the larger distance ran in the same amount of time. These results were expected as increasing the energy the body is able to use, should result in greater distance traveled. This shows the importance in runners being able to take in as many calories during a race as possible. A runner can increase the amount of calories they can consume during a race by taking nutrition that is easier to digest, taking water with the nutrition so that the salinity balance of the gut is kept, and practicing taking in nutrition during long training runs.

The last nutrition strategies we considered were, *s*_6_, *s*_7_, where the runner takes eleven and twenty-four 100 calorie gels throughout the race, respectively. These scenarios are unlikely to occur as a runner would struggle to consume this many gels without getting sick. We completed simulations with nutrition strategies *s*_6_ and *s*_7_ to see if the model would organically capture the effect of a runner taking in too much nutrition. As seen in the results from Table 3, this effect was not captured and the simulations simply show an increase in energy as more gels are added, and no negative side effects. In the simulation where the runners consumes 11 gels, they are able to run 46.0 km, while in 24 gel simulation the runner travels 52.9 kms. Simply put, with a fixed time race, we see an increase in distance traveled for every gel that is taken. Taking in eleven and certainly twenty-four 100 calorie gels throughout a race would most certainly upset the runners stomach and all of those carbohydrates would struggle to make it to the muscles due to imbalances in the stomach. The body does not handle the amount of accumulated energy in the nutrition compartment without a negative reaction. In future work we plan to handle this issue.

Table 4 shows the distances achieved using different VLa types across two different nutrition strategies. The VLa types labeled as “good", “average", and “bad" correspond to the glyc functions shown in Figure 4. For these runs, we tested the distance for the case where the runner takes in no nutrition during the race, as well as the runner taking in four 100 calorie gels.

**Table 4:** Total distance achieved by runners with different VLa types across two nutrient strategies.

**VLa type**	**Nutrition**	**Distance (km)**
Good	0 calories taken	41.7
Average	0 calories taken	40.0
Bad	0 calories taken	37.8
Good	Four 100 calorie gels	44.1
Average	Four 100 calorie gels	42.6
Bad	Four 100 calorie gels	40.5

In the three different simulations with varying VLa's, the runner consumes four 100 calorie gels during the race. In these results, the importance of runners training their body's to have lower maximum rate production of lactate for a lo ng distance runner is evident. The runner with the good VLamax type (i.e. the runner whose *glyc* function was made to use fewer carbohydrates than fats than the runners with average or bad VLa's) is able to run much further in the same amount of time. There is an 8.9–10.3% improvement in the runner with a good VLamax as compared with a bad VLamax depending on if the runners took in nutrition as well. We see the same race structure regardless of the VLa type, with both simulations showing the runners ending with ~0 glycogen energy, but similarly to the simulation where the runner takes more nutrition, the runner with the higher VLamax is able to maintain a higher force. The runner with a good VLamax can maintain a force of 1.97 × 10^4^ m/min^2^ throughout the race, while the runner with a bad VLamax was only able to maintain a level force of ~1.81 × 10^4^ m/min^2^. Some of these results can be seen in Table 5.

**Table 5:** Total distance achieved with different levels of runners and four lengths of time.

**Race time (min)**	**VVO2max (m/min)**	**Weight (Kg)**	**VLa type**	**Nutrition (Cals)**	***E*_*G*_(0) (KJ/Kg)**	**Dist. (Km)**
155	320	73	Avg	4 100 cal gels	150	42.9
155	320	73	Avg	0 100 cal gels	150	40.1
155	320	73	Good	4 100 cal gels	150	44.4
155	320	73	Bad	4 100 cal gels	150	41.6
180	250	80	Avg	0 100 cal gels	140	41.0
180	250	80	Avg	4 100 cal gels	140	42.7
215	200	80	Bad	0 100 cal gels	140	41.6
210	200	80	Avg	4 100 cal gels	144	44.3

To see optimal race strategies for individuals of all levels, we also completed scenarios including varying: the length of race, *T*, the runners mass, *m*, the initial glycogen energy, *E*_*G*_(0), and the runner's VVO2max. We use many of the same parameters from Table 2, but change the weight of the runner, *m*, length of race, *T*, as well as the VVO2max of the runners. Note that *c*_3_ and *M* also vary, but do so due to their relationship with *m* and *T*, respectively. Runners of different levels will finish the marathon in different lengths of time, thus the need to vary *T*. Also, runners of different abilities can have significantly different VVO2max values and VLa types, and therefore change depending on the individual. Table 5 shows the distances achieved for our different levels of runners over 4 time intervals.

To see the versatility of the model for individuals with different VLamax and VO2max inputs, we use the same parameters from Table 2, but vary the glyc function when analyzing the impact of Vlamax and vary the VVO2max, along with associated parameters, when analyzing the impact of VO2 max.

For runners who are running for 155 and 180 min with VVO2max's of 320 and 250, respectively, the results are realistic. The runners run at around 85% of their VO2max which is the suggested level by experts, and the impact of taking in-race nutrition compared without nutrition is substantial as expected. The runner who takes 180 min to finish the race and has an average VLamax has a 4% improvement when they take nutrition vs. when they do not. One issue can be found in the result from the scenario where the runner had a race time of 215 min and a bad VLA type (Table 5), as these results show this runner to be running at an unrealistically high percentage of their VO2max. This is most likely due to the fact that our model does not penalize a runner for running too fast, as long as they have enough glycogen energy. Recall, that our only constraints are that the energies have to be above 0, and that there are some upper bounds on forces and velocities. As the average finish time for a marathon such as the Berlin marathon is between 4 and 5 h (43), it is important for future work to address this. We plan to amend our current model such that the runner is not only being constrained by total amount of glycogen energy, as this limitation impacts the models accuracy for runners moving at slower velocities, as well as the model's ability to predict realistic optimal finishing times for shorter races.

## 4. Conclusions and limitations

To understand the impact of nutrition we formulated our model such that in-race nutrition could be accounted for in the energy differential equations. We created a marathon model described by velocity, fat energy, glycogen energy, and in-race nutrition differential equations. This marathon model better represented the body's energy systems and included nutrition pulses throughout the race. After formulating an optimal control problem with this system of differential equations, and with the goal to maximize the distance achieved over a fixed time interval, we proved the existence of an optimal control. We determined a discretized approximate solution to the associated optimal control problem using MATLAB's fmincon optimization software, that included a bounded variation penalty on our control force. We then optimized over a finite set of in-race nutrition strategies, obtaining a solution to our model for each strategy.

We analyzed the complicated role that in-race nutrition input has on a marathon runner as well as energy allocation dependent on the percentage of one's VO2max during running. Expressing the dynamics of the body's energy throughout a running race as two differential equations, that represent fat energy and glycogen energy, with usage determined by an allocation function dependent on the ratio of one's velocity compared to their VVO2max, is a valuable contribution to the field of runner models. Prior to our model, all energy dynamics in runner models, were based on available oxygen per unit mass, which does not allow for one to include in-race nutrition energy. As a runner has a much smaller storage supply of glycogen than fat, and the body prefers to use the glycogen at high levels of exertion, the in-race nutrition consists of essentially only carbohydrates. These factors required us to split up the energy into the two compartments and to use the allocation function. While we obtained an optimal solution for an expert runner, we also individualized our model such that it can be used to determine a race strategy for any type of runner as long as they know their weight, VO2max and VLamax type.

To obtain our approximate solution to the optimal control problem of maximizing distance for a fixed time, we discretized our problem, and then optimized, using Matlab's optimizing tool, fmincon. We included a penalization for variation in the objective function. The variation penalization was extremely important and the solution structure was very sensitive to this penalization. With too small of a variation penalty coefficient, the runners purposive force was often erratic and non-optimal. We found that the optimal trajectory for marathon runners was to run at a steady state velocity for the majority of the race and take in at least four 100 calorie supplements spread evenly throughout the event. Over a variety of runner's individual parameters, this racing strategy was optimal. A runner can improve their performance with consistent and targeted training to improve their VV02max and their VLamax, (which correlates to having a better *glyc* function) which can in turn drastically improve the their performance potential. When we approximated a solution to our problem for a runner with the parameters of the current world record holder in the marathon, our approximation was quite good with an error of 0.4%.

Our contributions to the field also include: formulating a system that includes energy allocation dependent on the ratio of their velocity and their velocity at VO2max, adding in-race nutrition to a runner model, and approximating a solution to the optimal control problem, amended to include a penalty bounding the variation of the control. As we have found that taking more carbohydrates throughout the race improves performance, it is natural to believe that one should take in as many carbohydrates as possible; however, this is unrealistic as the stomach's digestion slows through a running race and also struggles to tolerate high volume of carbohydrates in the form of glucose and fructose. Our initial marathon model does not account for this fact and our results showed that a runner's performance increased linearly with each gel they consumed, over the set of reasonable nutrition scenarios that were tested. To account for this limitation requires additional work, which will be presented in our next paper.

Upon executing different scenarios for our marathon model, we realized that our model was not appropriate for races between a sprint and a half marathon. In these scenarios we obtained results that were much faster than has been accomplished by humans thus far. These results came from only having energy constraints in model, when in reality, not all the energy in the body is available for use, due to the dependence on lactate production. An extended model with the effects of lactate will be presented in our followup paper. We also could add in some components from Kim et al. (9) for a more granular approach that includes tissue/organ subsystems and hormonal controllers to predict glucose homeostasis during moderate intensity exercise.

We view our model as a strong building block on which to add key features. We acknowledge some limitations of our model and the possibility to include more features like race terrain, temperature, humidity, and even loss of body weight during the race (44–47). As an important next extension, we would like to incorporate a key feature, hydration to our model. Hydration levels and fluid overload can be a huge factor for a runner (43, 47–49). Low hydration can negatively affect the rate at which the body uptakes the nutrition. One possibility could be changing our nutrition uptake function to represent low, medium, or high levels of hydration. Building this feature in would be the next step in advising runners on optimal intake during running races. This work does not aim to capture every related race variable, but will hopefully be a building block for future runner models.

## Data availability statement

The original contributions presented in the study are included in the article/Supplementary material, further inquiries can be directed to the corresponding author.

## Author contributions

CC, SL, GC, and WH contributed to conception and design of the study. CC, SL, and WH performed the mathematical analysis. CC wrote the first draft of the manuscript. All authors contributed to manuscript revision, read, and approved the submitted version.
