# Supplementary material for: Optimally controlling nutrition and propulsion force in a long distance running race
Source: Front Nutr. 2023 May 18;10:1096194. doi: 10.3389/fnut.2023.1096194 (PMC10233029; doi:10.3389/fnut.2023.1096194)
Supplement: Supplementary file 1 [file Presentation_1.pdf]

Supplementary Material

1 SUPPLEMENTARY DATA

1.1 Existence of the optimal control

THEOREM 1. *Given a nutrition strategy s_i, there exist an optimal control f^* that solves the optimal control problem of maximizing $J_i(f) = \int_0^T V(t)dt$ with corresponding state equations over admissible controls,*

$$\mathcal{U} = \{f \in L^2(0,T) \,|\, 0 \leq f(t) \leq f_{max}, a.e., \; E_F(t) \geq 0, \; E_G(t) \geq 0, \, for \, t \geq 0\}.$$

PROOF. First, the admissible control set is nonempty since the control $f = 0$ satisfies the conditions. Our objective functional, $J(f)$ is uniformly bounded due to bounds on the states and controls. There exists a maximizing sequence of control functions denoted by f^n such that

$$\lim_{n\to\infty} J(f^n) = \sup_{f\in\mathcal{U}} J(f),$$

and a corresponding sequence of states V^n, E_F^n, E_G^n, N^n. Note that the control sequence and the state sequences are uniformly bounded. By the Banach-Alaoglu theorem (2), there exists a control f^* such that on a subsequence $f^n \rightharpoonup f^*$ weakly in $L^2(0,T)$. From the state differential equations we see that the derivative sequences are also uniformly bounded. Thus, V^n, E_F^n, E_G^n, N^n are uniformly Lipschitz and thus equicontinuous. Therefore by the Arzela-Ascoli theorem, on a subsequence, we have uniform convergence of our corresponding state functions

$$V^n \to V^*, E_F^n \to E_F^*, E_G^n \to E_G^*, N^n \to N^* \; .$$

We need to show that f^* is an optimal control and V^*, E_F^*, E_G^*, N^* are corresponding optimal states. To show those states correspond to control to f^*, we illustrate this by showing the E differential equation:

$$E_F^n(t) - E_F(0) = \frac{-1}{1000}\int_0^t f^n V^n (1 - glyc(V^n))ds$$

and

$$|\int_0^t \frac{f^n V^n}{1000}(1 - glyc(V^n))ds - \int_0^t \frac{f^* V^*}{1000}(1 - glyc(V^*))ds|$$
$$\leq \; ||f^n||_2||V^n - V^*||_2 + |\int_0^t V^*(f^n - f^*)ds| \quad \text{(by Holder's Inequality)}$$

We have that all 4 states correspond to the control f^* . Next, f^* is an optimal control since,

$$\lim_{n\to\infty} J(f^n) = \lim_{n\to\infty}\int_0^T V^n dt = \int_0^T V^* dt = J(f^*) \; .$$

Now that we've proven an optimal control exists, we begin solving the optimal control problem using Pontryagin's Maximum Principle (PMP) (6) by determining the set of necessary conditions, using the Hamiltonian with state constraints. We solve the maximum distance problem, obtaining an f^* for each nutrition strategy, s_i, and maximize over our set of finite strategies S.

1.2 Necessary Conditions for the Maximum Distance Problem

We determine the necessary conditions by using PMP (6) to compute the Lagrangian, which is the Hamiltonian with state constraints:

$$\mathcal{L} = V + \lambda_1(f(t) - \frac{V}{\tau}) + \lambda_2(-af(t)V(1 - glyc(V))) + \lambda_3(c_3 j(N) - af(t)V glyc(V)) \\ + \lambda_4(s(t) - dN - j(N)) + \eta E_G \ ,$$

where the penalty function $\eta(t) \geq 0$ is a Lagrangian multiplier appended to E_G as a state constraint penalty. Note that we do not have a state constraint penalty for E_F as E_F will not get close to 0 over the time interval. The function η satisfies: $\eta \equiv 0$ where $E_G^* > 0$, and $\eta \geq 0$ otherwise when the state constraint is tight (i.e. $E_G = 0$) Since we seek to maximize $\mathcal{L}$ with respect to $f(t)$, a state variable violating the constraint would decrease $\mathcal{L}$ and not be optimal as $\eta E_G < 0$.

Also, PMP(6) gives the existence of adjoint functions , λ_1, λ_2,λ_3 and λ_4, satisfying system (S1) - (S3) appending the state equations to the Hamiltonian.

$$\lambda_1' = -\frac{\partial \mathcal{L}}{\partial V} = -(1 - \frac{\lambda_1}{\tau} - \lambda_2 af(t) + \lambda_2 af(t)V\frac{\partial glyc}{\partial V} + \lambda_2 f(t)a glyc(V) - \\ \lambda_3 af(t)V\frac{\partial glyc}{\partial V} - \lambda_3 af(t)glyc(V)) \tag{S1}$$

$$\lambda_2' = -\frac{\partial \mathcal{L}}{\partial E_F} = 0 \ , \ \lambda_3' = -\frac{\partial \mathcal{L}}{\partial E_G} = -\eta \tag{S2}$$

$$\lambda_4' = -\frac{\partial \mathcal{L}}{\partial N} = -(c_3 c_4 \lambda_3 - \lambda_4 d - \lambda_4 c_4) \tag{S3}$$

with transversality conditions $\lambda_1(T) = 0$, $\lambda_2(T) = 0$, $\lambda_3(T) = 0$, $\lambda_4(T) = 0$

For $t \in [0, T]$, due to it's initial condition, E_F, stays positive for any choices of $f(t)$ in the admissible control set and thus $\lambda_2 = 0$ over $[0, T]$.

Since our objective functional and state equations are linear in the control, we consider the different signs of the derivative of $\mathcal{L}$ with respect to $f(t)$, which is the switching function $\phi(t)$.

$$\phi(t) = \frac{\partial \mathcal{L}}{\partial f} = \lambda_1 - \lambda_3 aV glyc(V) \tag{S4}$$

At time t, for $\phi(t) < 0$ we will have a maximum when $f^*(t) = 0$, whereas for $\phi(t) > 0$ we will have a maximum when $f^*(t) = f_{max}$. However, PMP (6) does not tell us what happens when $\phi(t) = 0$. More information with respect to control f than $\frac{\partial \mathcal{L}}{\partial f} = 0$ is needed when $\phi(t) = 0$ on a subinterval. If this

happens only for for finite number of time points, we would have a bang-bang control and those points would represent the switching times. On the other hand, if $\phi(t) = 0$ on a subinterval of time, then we would have a singular control.

For optimal control problems with state constraints there is the possibility of boundary singular sub-arcs as well as interior singular sub-arcs. Boundary singular sub-arcs occur when the constraint is tight, meaning when $E_G(t) = 0$. If $E_G(t) = 0$, that implies $E'_G = 0$, and solving the glycogen energy differential equation to obtain $f_{s,b}$, provided that $V(glyc(V)) \neq 0$. Note that as $glyc(V)$ accounts for the percent of glycogen energy being used and there is always a percentage of fuel usage coming from glycogen, this function will never be 0. Due to the structure of the differential equation for V, as soon as f is positive, V is also positive. Hence, $V(glyc(V)) \neq 0$ when the boundary singular arc is active.

If the constraint is not tight, then we have the possibility of an interior singular sub-arc that we can obtain by differentiating the switching function with respect to time twice, noting that on an interior singular sub-arc $\eta(t) = 0$, and solving for $f_{singular}$.
When the constraint $E_G(t)$ is not tight, $\eta(t) = 0$; however, when it is tight we solve for it using the fact that over the small time interval where the glycogen energy constraint is tight, the switching function, $\phi(t)$, is 0 on a boundary subarc and thus also $\phi'(t) = 0$. We are then able to solve this equation for $\eta(t)$ to get our complete characterization of $\eta(t)$.

For our last necessary condition we have that the Transversality Conditions: $\lambda_1(T) = \lambda_2(T) = \lambda_4(T) = 0$.

From the above, our optimal control, force, has the following structure:

$$f(t) = \begin{cases} 0 & for \quad \phi(t) < 0 \\ f_{singular}(t) & for \quad \phi(t) = 0 \;\; E_G \neq 0 \\ f_{s,b}(t) & for \quad \phi(t) = 0 \;\; E_G = 0 \\ f_{max} & for \quad \phi(t) > 0 \end{cases}$$

This $f(t)$ represent the force profile of which a runner should adhere to in order to run the optimal race. We have shown that our problem meets all of the necessary conditions for existence of a singular interior sub-arc, including the GLC condition (3) (not shown), which suggests that the optimal trajectory could include a singular component. To summarize the control trajectory, we know that the optimal control begins with a maximum force sub-arc, that for a race with large enough T, an optimal control must be comprised of more than just a maximum force arc, that a singular boundary force sub- arc exists (and likely at the end of the time interval), that a singular interior sub-arc is likely, and that intuitivly, it is unlikely for a zero force arc to exist, as that would not be optimal.

We believe that for our system, the control is singular for the majority of the event, and is comprised of a maximum force sub-arc, followed by a singular interior sub-arc, and finishing with a singular

boundary sub-arc, as has been shown to be the trajectory in simpler systems in (4; 5; 1; 7). Unfortunately one is unable to easily obtain an exact solution structure with switching times.

REFERENCES

Aftalion, A. and Bonnans, J. F. (2014). Optimization of running strategies based on anaerobic energy and variations of velocity. SIAM Journal on Applied Mathematics, 74(5):1615-1636.

Friedman, A. (1982). Foundations of Modern Analysis. Dover Books on Mathematics Series. Dover.

Robbins, H. M. (1967). A generalized Legendre-Clebsch condition for the singular cases of optimal control. IBM Journal of Research and Development, 11(4):361-372.

Keller, J. B. (1973). A theory of competitive running. Physics Today, 26(9):43-47.

Pitcher, A. B. (2009). Optimal strategies for a two-runner model of middle-distance running. SIAM Journal on Applied Mathematics, 70(4):1032-1046.

Pontryagin , L. S., Boltyanskiii, V. G., Gamkrelize, R. V., and Mishchenko, E. F. (1962) The Mathematical Theory of Optimal Processes, Wiley.

Woodside, W. (1991). The optimal strategy for running a race (a mathematical model for world records from 50 m to 275 km). Mathematical and Computer Modeling, 15(10):1- 12.
